# The Association between Failure to Thrive or Anemia and Febrile Seizures in Children between 6 Months to 6 Years Old Age

**Published:** 2018

**Authors:** Fahimeh SOHEILIPOOR, Azita TAVASOLI, Zeinab BABASAFARI RENANI

**Affiliations:** 1 1.Pediatric Endocrinology, Rasool- e Akram hospital, Iran University of Medical sciences, Tehran, Iran.; 2 2.Pediatric Neurology Department , Ali-Asghar Children’s Hospital, Iran University of Medical sciences, Tehran, Iran.; 3 3.General physician. Ali-Asghar Children’s Hospital, Iran University of Medical Sciences, Tehran, Iran.

**Keywords:** Anemia, Failure to thrive, Febrile seizure

## Abstract

**Objectives:**

Febrile seizure is the most common seizure disorder in childhood. Anemia or failure to thrive can predispose children to febrile seizure by affecting the nervous system function. The current study investigated the association between febrile seizures and anemia or failure to thrive.

**Materials and Methods:**

This case-control study was performed on 307 children 6 months to 6 yr old age hospitalized at the Ali Asghar Children`s Hospital, Tehran, Iran from 2011 to 2014 divided into two groups as follows: A case group including 158 children with febrile seizures and a control group including 149 febrile children without seizure. The amount of Hgb, Hct, RBC count, MCV, MCH, and MCHC was recorded and weight-for-age and weight-for-height was calculated based on the WHO Z-Score charts. The data were compared between two groups.

**Results:**

There were no differences regarding age and sex between the groups. Statistically significant differences were found regarding the mean RBC count between the case group (4.38×10^6^ ± 0.72×10^6^) and the control group (4.24×10^6^ ± 0.84×10^6^) (*P*=0.013), as well as about the mean MCV that was 78.73 ± 0.97 and 76.78 ± 1.00 in the case and control groups, respectively (*P*=0.005). Anemia was seen in 28.5% of the cases and 42.3% of control group which was statistically significant (*P*=0.012). There was not statistically significant difference regarding failure to thrive between two groups.

**Conclusion:**

In children with febrile seizures, anemia was lower comparing with febrile children without seizure. Moreover, there was not any association between failure to thrive and febrile seizures.

## Introduction

Febrile seizure is the most common seizure disorder during infancy and childhood and its incidence varies from 2%-14% in different populations (1). It occurs in the children older than one-month age with a febrile illness and without any central nervous system infection, acute electrolyte abnormality, or history of afebrile seizures (1). Some known risk factors of febrile seizures include positive family history of febrile seizures, attending the nursery for more than one month, developmental delay, going to kindergarten, sudden increase in body temperature, and maternal smoking (2,3). 

Failure to thrive (FTT) can have different effects on the growing children such as secondary immunodeficiency, permanent damage to several parts of the central nervous system, and cognitive as well as developmental dysfunction (4). Its diagnosis will be raised if the child’s physical growth is significantly lower than peers that is when the growth level is below the 3^rd^ or 5^th^ percentile or there is a drop in the growth rate to cut two percentile curves in a short time (4,5). The prevalence of growth retardation depends on the sample population. In developed countries, 5%-10% of premature children and children with the lower socioeconomic status can have growth disorders. These statistics is much greater in developing countries with high rates of malnutrition and HIV infection (4,5). Trace elements affect many pathways and specific enzymes such as oxidative system in the central nervous system. Weakening of the antioxidative defense mechanisms and increased levels of free radicals can result to seizures (6). Thereby, electrolyte imbalances and deficiency of micronutrients such as zinc, selenium, magnesium, copper and vitamin D seen in the malnutrition and failure to thrive can predispose children to the febrile seizures (7–10).

Iron plays an important role in the neural activity, enzymatic reactions and metabolism of neurotransmitters (11). In developing countries, iron deficiency is the most common nutritional problem. its peak incidence is in the ages between 6 to 24 months old, which overlaps with the peak incidence of febrile convulsion that is in the ages from 14 to 18 months old (11,12). In these countries, 46%-66% of children less than four yr age have anemia that half of this prevalence is due to iron deficiency anemia (13).

Anemia- defined as hemoglobin level below two standard deviations from normal values for age- affects the developing brain via changing hippocampal neuronal evolutionary mechanisms, energy metabolism dysfunction, delayed development of myelin, blunting the visual and auditory action potentials, and changes in synaptic neurotransmitter systems including norepinephrine, dopamine, glutamate, and gamma-aminobutyric acid (GABA) (14,15). The possibility of lowering seizure threshold and increasing the risk of febrile seizures by the anemia have been discussed in many studies (13,14). While others have concluded that anemia does not predispose to febrile seizures and may even have a protective effect (16–18).

We investigated the relationship between the anemia or failure to thrive and febrile seizures to find out whether these prevalent health conditions could predispose the children to febrile seizures or not.

## Materials and Methods

This case-control study was conducted on the records of 307 children aged from 6 months to 6 yr, admitted to the Ali Asghar Children`S Hospital, Tehran, Iran from 2011 to 2014. They were divided into a case group including 158 children admitted with febrile seizures and a control group including 149 children with an acute non complicated febrile disease without febrile seizure such as respiratory or urinary tract infections, gastroenteritis, otitis media and nonspecific viral infections. Patients with febrile diseases that were more suspicious to have anemia such as chronic cardiovascular, renal, metabolic, rheumatologic, and malignant diseases, hemoglobinopathies and other blood disorders, any evidence of CNS infections or organic causes of seizure, and history of afebrile seizures, epilepsy, or anti-convulsive treatments were excluded. Two groups were matched regarding age, sex, body temperature, developmental status, feeding type and history of taking iron supplements. 

Simple febrile seizure was defined as a non-focal single seizure lasting less than 15 min associated with a core temperature of at least 38 °C in the absence of any evidence of CNS infections or other causes of seizure. Complex febrile seizure was defined as a focal seizure or seizure episodes lasting longer than 15 min, or seizure episodes occurring more than once in 24 hours. Anemia was defined as hemoglobin level below two standard deviations from normal values for age, i.e., Hgb < 10.5 gr/dl for children aged 6-24 months and Hgb<11.5 g/dl for 2-6 yr old. Failure to thrive was defined in two ways: it was once diagnosed when Z-Score of weight-for-height was less than -2 and once when Z-Score of weight-for-height or Z-Score of weight-for-age was less than -2 according to standardized WHO Z-Score growth charts. Axillary temperature more than 38 °C (when 0.5 °C was added to it) was defined as fever in two groups. 

The Ethics Committee of Iran University of Medical Sciences approved this study.

Variables including age, gender, weight, height, cause of fever, family history of febrile seizures, history of developmental delay, seizure type (simple or complex), RBC count, hemoglobin, hematocrit, MCV, MCH, and MCHC were collected. Data were analyzed by SPSS version 22 (Chicago, IL, USA). Qualitative variables were analyzed by chi-square tests and mean values were compared using independent *t*-test. *P* values less than 0.05 were considered significant.

## Results

The patients included 177 boys and 130 girls with a mean age of 21.9 ± 34.3 months (*P*> 0.05) (Table 1). Among 158 patients in the case group, 109 (69%) had simple and 49 (31%) complex febrile seizure.

The most common causes of fever in all of the patients were respiratory tract infections (41.4%) and gastroenteritis (21.5%) (Table 2). Family history of febrile seizures was positive in 61 patients (38.6%) in the case and only 2 patients in the control group (1.3%) which differs significantly (*P*< 0.001). There were 10 patients in the case group (6.32%) and 4 in the control group (2.68%) with developmental delay but the difference was not significant (*P*=0.172). In addition, from these 10 patients of the case group, 4 patients (40%) had simple seizure and 6 (60%) complex seizure (*P*=0.071). Among 49 patients with complex febrile seizure, 26 (53%) were male (*P*=0.73).

There were significant differences between two groups regarding mean RBC count (4.38 × 10^6^ ± 0.72 × 10^6^ in the case versus 4.24 × 10^6^ ± 0.84 × 10^6^ in the control group) (*P*=0.013) and the mean MCV (78.73 ± 0.97 in the case versus 76.78 ± 1.00 in the control group ) (*P*=0.005) (Table 3). From the 108 anemic patients, 45 (28.5%) were in the case and 63 (42.3%) in the control group (*P*=0.012). In the patients with complex febrile seizure, 17 (34.7%) were anemic and 32 (65.3%) were not, but the difference was not significant (*P*=0.258).

Considering patients with Z-Score of weight-for-height less than -2 as the patients with failure to thrive (FTT1), 20 children (6.5%) had failure to thrive including 7 patients (4.43%) in the case and 13 (8.72%) in the control group which did not show significant difference (*P*=0.166). Considering patients with Z-Score of weight-for-height or Z-Score of weight-for-age less than -2 as patients with failure to thrive (FTT2), 32 children (10.4%) had failure to thrive including 11 patients (6.96%) in the case and 21 (14.09%) in the control group which did not show significant difference as well (*P*=0.060). Comparison of Z-Scores of weight-for-age (*P*=0.082), height-for-age (*P*=0.954), weight-for-height (*P*=0.227), and BMI-for-age (*P*=0.437) between the groups showed no significant differences.

**Table 1:** Mean age and gender distribution in the two groups

	Case group N (%)	Control group N (%)	Total N (%)	*P* value
Mean age (month)	22.3 ± 22.9	21.6 ± 38.6	21.9 ± 34.3	0.162
Male patients	88 (55.7%)	89 (59.7%)	177 (57.7%)	-
Female patients	70 (44.3%)	60 (40.3%)	130 (42.3%)	0.49

**Table 2:** Causes of fever in the two groups

	Case group N (%)	Control group N (%)	Total N (%)
Respiratory tract infections	81 (51.3)	46 (30.9)	127 (41.4)
Gasteroenteritis	30 (19)	36 (24.2)	66 (21.5)
Otitis media	8 (5.1)	3 (2)	11 (3.6)
Sinusitis	_	9 (6)	9 (2.9)
Urinary tract infection	7 (4.4)	8 (5.4)	15 (4.9)
FWLS¹	_	28 (18.8)	28 (9.1)
Not known	32 (20.1)	19 (12.7)	51 (16.6)

1 : fever without localizing sign

**Table 3:** Mean values of hematologic parameters in the two groups

	Case group	Control group	*P* value
RBC count (million / μl)	4.38×10^6^ ± 0.72×10^6^	.24×10^6^ ± 0.84×10^6^	0.013
Hgb (gr / dl)	11.26 ± 0.17	11.13 ± 0.24	0.371
Hct (%)	33.45 ± 0.46	33.36 ± 0.65	0.823
MCV (fl)	78.73 ± 0.97	76.78 ± 1.00	0.005
MCH (pg)	26.39 ± 0.41	25.88 ± 0.40	0.082
MCHC (gr / dl)	33.7 ± 0.29	33.41 ± 0.27	0.156
Anemia (%)	28.5%	42.3%	0.012

## Discussion

Results of our study showed that the prevalence of anemia in the febrile seizure group was significantly lower than the control group (28.5% versus 42.3%). This is identical with the results of another study that evaluated 51 febrile children 6-36 months old age in two groups (with and without seizure) for their iron status. Iron deficiency was less frequent in febrile seizure group. They suggested a probable relationship between iron deficiency and 7.8 fold decreased risk of febrile seizure (19). They stated that brain lipid peroxidation induced by iron may lead to febrile seizure. Moreover, cell membrane uptake and discharge of dopamine, gamma aminobutyric acid and other neurotransmitters may be affected by iron. Thereby, the seizure threshold may be increased due to iron deficiency (19). Although, a limited factor in their study was the small sample volume, the patients were well matched in the respect of related variables. In comparison, we have studied much more patients in a more wide range of age and have match their interfering factors appropriately. 

In another study conducted on 120 patients between 6 months to 5 yr old, Talebian et al. showed anemia in 12% of patients with febrile seizure versus 20% of febrile patients without seizure and concluded that anemia might protect patients against febrile seizure (20). Derakhshanfar et al. in their study on 1000 children, showed that febrile seizure was less frequent in children with iron deficiency anemia comparing with other children (21). They noted that iron deficiency causes decrease in the level and activity of the exciting neurotransmitters including monoamine oxidase and aldehyde oxidase and leads to a reduction in excitation of the neurons and seizures. In addition, Yousefichaijan et al. in a cross-sectional study carried out on 382 children aged between 6 months-6 yr found the blood indices in patients with febrile seizure were significantly higher than the febrile patients without seizure were (22). They stated that iron deficiency anemia probably could protect patients against febrile seizure by raising the threshold of seizure. The results of other studies have discrepancy with ours (23–25). They notified that fever can aggravate the noxious effects of anemia on the brain and leads to seizure. Miri-Aliabad et al. reported no relationship between anemia and febrile seizures in their study (26).

In respect of the well-known influences of micronutrients on febrile seizures, malnutrition and FTT may increase the risk of febrile seizures through the lack of micronutrients such as zinc, magnesium, selenium and vitamin D. Yilmaz and Balci showed that serum selenium levels were significantly lower in the simple febrile seizure patients. They stated oxidative stress and low function of antioxidative mechanisms induce seizures by production of free radicals and selenium prevent oxidative injury through specific enzymes and therefore might prevent seizures (6). Afshar et al. **showed** in febrile seizure patients serum level of magnesium was lower comparing with febrile patients without seizure, and proposed magnesium might affect in developing seizure in them (10). Nasehi et al. in a systematic review concluded that decreased serum level of zinc could be a predicting factor for febrile seizure (27). In spite of, we did not find any significant differences between groups using two FTT definitions (FTT1 and FTT2). As recurrent infections is one of the clinical presentations of failure to thrive (4), collecting a control group of febrile children with considering this confounding factor could be more accurate in studying influence of FTT on febrile seizure.

Our study demonstrated that anemia was seen less in children with febrile seizures. In addition, no relation was found between failure to thrive and febrile seizures.

Although in most studies on febrile seizures, as well as the current study, the control group had been selected from hospitalized patients with a febrile illness without convulsions, it seems that choosing the control group from outpatient febrile children with simple infectious diseases could provide a more reliable control group and more accurate results therefore.

**In conclusion,** according to contradictory results about the association between anemia and febrile seizures in various studies and the lack of enough studies regarding the effect of FTT on febrile seizures, further prospective and precise investigations in this respect is recommended.
